# Combining structured and unstructured data in EMRs to create clinically-defined EMR-derived cohorts

**DOI:** 10.1186/s12911-021-01441-w

**Published:** 2021-03-08

**Authors:** Charmaine S. Tam, Janice Gullick, Aldo Saavedra, Stephen T. Vernon, Gemma A. Figtree, Clara K. Chow, Michelle Cretikos, Richard W. Morris, Maged William, Jonathan Morris, David Brieger

**Affiliations:** 1 Centre for Translational Data Science, The University of Sydney, Sydney, Australia; 2 Northern Clinical School, The University of Sydney, Sydney, Australia; 3 Susan Wakil School of Nursing and Midwifery, The University of Sydney, Sydney, Australia; 4 Faculty of Health Sciences, The University of Sydney, Sydney, Australia; 5 Cardiothoracic and Vascular Health, Kolling Institute of Medical Research and Department of Cardiology, Royal North Shore Hospital, Northern Sydney Local Health District, Sydney, Australia; 6 Westmead Applied Research Centre, The University of Sydney, Sydney, Australia; 7 Department of Cardiology, Westmead Hospital, Sydney, Australia; 8 Clinical and Population Perinatal Health, Northern Sydney Local Health District, Sydney, Australia; 9 Centre for Population Health, NSW Ministry of Health, Sydney, Australia; 10 Department of Cardiology, Central Coast Local Health District and University of Newcastle, Sydney, Australia; 11 Department of Cardiology, Concord Hospital, Sydney, Australia

**Keywords:** Electronic medical record, Cohort identification, Electronic phenotype, Acute coronary syndrome

## Abstract

**Background:**

There have been few studies describing how production EMR systems can be systematically queried to identify clinically-defined populations and limited studies utilising free-text in this process. The aim of this study is to provide a generalisable methodology for constructing clinically-defined EMR-derived patient cohorts using structured and unstructured data in EMRs.

**Methods:**

Patients with possible acute coronary syndrome (ACS) were used as an exemplar. Cardiologists defined clinical criteria for patients presenting with possible ACS. These were mapped to data tables within the production EMR system creating seven inclusion criteria comprised of structured data fields (orders and investigations, procedures, scanned electrocardiogram (ECG) images, and diagnostic codes) and unstructured clinical documentation. Data were extracted from two local health districts (LHD) in Sydney, Australia. Outcome measures included examination of the relative contribution of individual inclusion criteria to the identification of eligible encounters, comparisons between inclusion criterion and evaluation of consistency of data extracts across years and LHDs.

**Results:**

Among 802,742 encounters in a 5 year dataset (1/1/13–30/12/17), the presence of an ECG image (54.8% of encounters) and symptoms and keywords in clinical documentation (41.4–64.0%) were used most often to identify presentations of possible ACS. Orders and investigations (27.3%) and procedures (1.4%), were less often present for identified presentations. Relevant ICD-10/SNOMED CT codes were present for 3.7% of identified encounters. Similar trends were seen when the two LHDs were examined separately, and across years.

**Conclusions:**

Clinically-defined EMR-derived cohorts combining structured and unstructured data during cohort identification is a necessary prerequisite for critical validation work required for development of real-time clinical decision support and learning health systems.

## Background

The widespread adoption of electronic medical records (EMR) offers unprecedented opportunities to rapidly ascertain and examine clinical data at large-scale and low cost; such information is essential for applications such as audit and feedback, near real-time clinical decision support as well as supporting research objectives through cohort studies, registries and large-scale pragmatic clinical trials [1–3]. Administrative coding systems such as International Classification of Diseases and Related Health Problems (ICD)-10 provide a translation of healthcare diagnoses, procedures, medical services, and medical equipment into universal codes [4], however do not provide a granular view of a patient’s presentation, severity of disease and clinical sequence during an episode of care [4–6] and have variable accuracy [7]. As such, improved computational methods which maximise the depth and accuracy of information extracted from production-level EMR systems are essential to fulfil the promise of real-time clinical decision support which rely on a reliable knowledge base to guide clinical decision making within a learning health system [8].

The development of robust methodologies that enable identification of clinical-defined cohorts from the overall patient population captured in production EMR systems (e.g. Cerner, Epic) are a critical first step [9]. The most straightforward approach is to use clearly defined events or procedure codes (e.g. type of surgery, cancer diagnosis) associated with the hospitalisation to identify cohorts [10, 11]. However, diagnostic codes alone are insufficient for identifying clinical EMR-derived cohorts due to strict rules adhered to for coding, underreporting and the complexity of diseases being assigned a single code [12]. The cohort identification process becomes even more challenging when diseases and conditions have heterogeneous aetiology and a spectrum of severity [13–15]. Approaches to data extraction from production EMR systems also have to consider whether there is ready access to production-level EMR environments (e.g. live systems, back-ups, copies of the production EMR etc.) which may limit the granularity and timeliness of information that is available for cohort identification as well as the opportunity for iteration during the cohort identification process.

There have been few reports describing how production EMR systems can be systematically queried to identify and reliably extract information from a clinically-defined cohort of interest and limited studies leveraging the > 70% of the EMR that is captured in free-text during this process [16–20]. Furthermore, previous studies have used structured data fields (e.g. ICD-10 code) to identify the population for data extraction which may lead to eligible patients being missed during data extraction if an ICD-10 code was absent or mis-coded [21, 22] or cohort identification has occurred *after* the data has been extracted from the production EMR system, which would also result in missed eligible patients [12, 23]. Mis-coding would lead to measurement error and missing data would contribute to selection bias and counteract the statistical power available from leveraging data housed in EMRs.

The aim of this study was to develop a generalisable methodology used within production EMR systems for creating high-fidelity clinically-derived EMR cohorts for complex diseases/conditions which cannot exclusively use diagnostic or procedure codes to identify a cohort of interest. Patients with possible acute coronary syndrome (ACS) were selected as an exemplar given its heterogeneous aetiology and spectrum of severity during presentation. This approach can be applied to other complex diseases/conditions including but not limited to mental illness, asthma, rheumatoid arthritis, chronic kidney disease [13–15, 19].

## Methods

### Source population

The eight metropolitan and seven rural/regional Local Health Districts (LHD) in New South Wales (NSW), Australia are responsible for managing public hospitals and health institutions and for providing health services within a geographical area. The source population presented to health care facilities in Northern Sydney LHD and Central Coast LHD, two of the eight metropolitan LHDs in NSW. Within Northern Sydney LHD and Central Coast LHD, there were eight publicly-funded hospitals that admitted patients with possible ACS. This included two tertiary hospitals with 24-h percutaneous coronary intervention capability and six referral hospitals; all of which used Cerner Millennium information systems. These two LHDs had a combined estimated resident population of 1.26 million people [24]. In this protocol paper, we followed the guidelines developed in the RECORD statement for reporting studies conducted using observational, routinely-collected health data [25].

### Study inclusion criteria and rationale

**Fig. 1:**
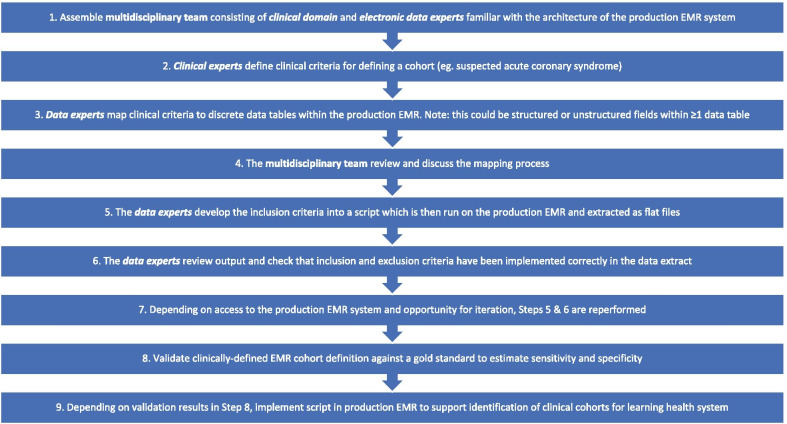
General methodology for creating clinically-defined cohorts using structured and unstructured data in production EMR systems

The methodology for cohort identification is summarised in Fig. 1. A multi-disciplinary team consisted of 5 clinicians (cardiologists, population health physicians, nurses) and 5 electronic data experts (data engineers, business analysts, data analysts, analytics translator). The multidisciplinary team varied in experience from early career through to professor level with career stages equally represented (Step 1, Fig. 1). In a series of initial meetings, the cardiologists defined clinical criteria to identify patients presenting with possible ACS (Step 2, Fig. 1). The data experts then mapped the clinical criteria to discrete data tables within the Cerner Millennium EMR system to locate the data elements required for identification of patients using these clinical criteria (Step 3, Fig. 1). This mapping process was discussed and agreed upon at iterative meetings over a 3-month period with the multi-disciplinary team (Step 4, Fig. 1). This approach of using a multi-disciplinary team to define clinical criteria which can then be mapped to discrete data tables in the EMR can be applied to any condition/disease of interest. During this process, we considered the previously described phenotype algorithm model workflow model for portable algorithms [26], against existing technological constraints for extracting data from Cerner EMR systems within our health jurisdiction (Steps 5–7, Fig. 1). The next steps would involve validation of a clinically-defined EMR cohort against a gold standard to estimate sensitivity and specificity for a diagnosis. Depending on whether the sensitivity and specificity results were deemed clinically acceptable, the script would then be implemented in a production EMR environment to support identification of clinical cohort for learning health systems (Steps 8–9, Fig. 1). The methods of such validation studies and implementation into production EMR systems are beyond the scope of the current study but are a critical step prior to implementation within learning health systems.

For the current study, seven inclusion criteria to identify possible ACS were developed to identify the cohort of interest (Table 1). If any of the seven inclusion criteria were met for an encounter during the study period, the encounter (termed an “*eligible*” encounter) was deemed eligible for inclusion into the study population. An encounter was defined as an electronically recorded interaction between a patient and healthcare provider, characterised by a unique identifier and admission time. Once an encounter met any of the seven inclusion criteria, all clinical, biochemical and demographic information contained within the EMR were extracted. This also included information from “*reference”* encounters for the individual patient dating back to 2002 when the EMR system was first implemented in the health district.

**Table 1:** Study inclusion criteria

Inclusion criteria	Definition
1	The “Reason for Visit” (free text field) for the presentation contained any of the ACS-related symptoms or keywords described in Additional file 1: Information Part 1
2	The patient was placed on a cardiac pathway care plan OR the information collected during emergency department triage (free-text), separate to the Presenting Information field above, contained any of the list of ACS-related symptoms or keywords (Additional file 1: Information Part 1)
3	Orders were placed in the EMR for a troponin test OR a 12 lead ECG OR for any of the following investigations: coronary angiogram, exercise stress test, stress echocardiogram, sestamibi scan, CT coronary angiogram, CT pulmonary angiogram
4	The patient’s EMR contained a “Cardiac Monitoring” form, meaning that the patient had been placed on a cardiac monitoring pathway
5	The patient had a result recorded in the EMR from a sestamibi scan, CT coronary angiogram, CT aortic angiogram or CT pulmonary angiogram
6	Any of the ICD-10 Australian Modification codes recorded for an encounter started with “I21”, “I22”, “I23”, “I24” or “I25” OR the episode of care had any of the following diagnoses (SNOMED CT) recorded in the EMR: “acute myocardial infarction”, “acute non-ST segment elevation”, “acute ST segment elevation”, “acute non-q wave infarction”, “angina”
7	The encounter contained a scanned 12-lead ECG image

For criteria (1) and (2), an initial list of ACS-related symptoms, keywords and abbreviations, for patients presenting with possible ACS was developed in consultation with the clinical reference group. The list was extended by manual review of ~ 50 Emergency Department triage forms in patients with an ICD-10 code for STEMI (I21.0, I21.1, I21.2, I21.3) or NSTEMI (I21.4). Next, we examined the frequency of each of the search terms for relevant data fields within a subset of ED triage forms obtained from a 3-month test extract (n ~ 30,000 encounters between 1/4/17 to 30/6/17). Given the unstructured nature of free-text, we also identified and included misspellings and abbreviations for each of the terms explicitly due to the text processing limitations of Cerner Command Language (CCL) used to extract the data; regular expressions were not able to be used in CCL. If the keywords were present at least 20 times (chosen as an arbitrary cut-off) in the subset of 30,000 ED Triage forms, they were then included in the final list of search terms of ACS-related symptoms and keywords.

### EMR data extraction

**Fig. 2:**
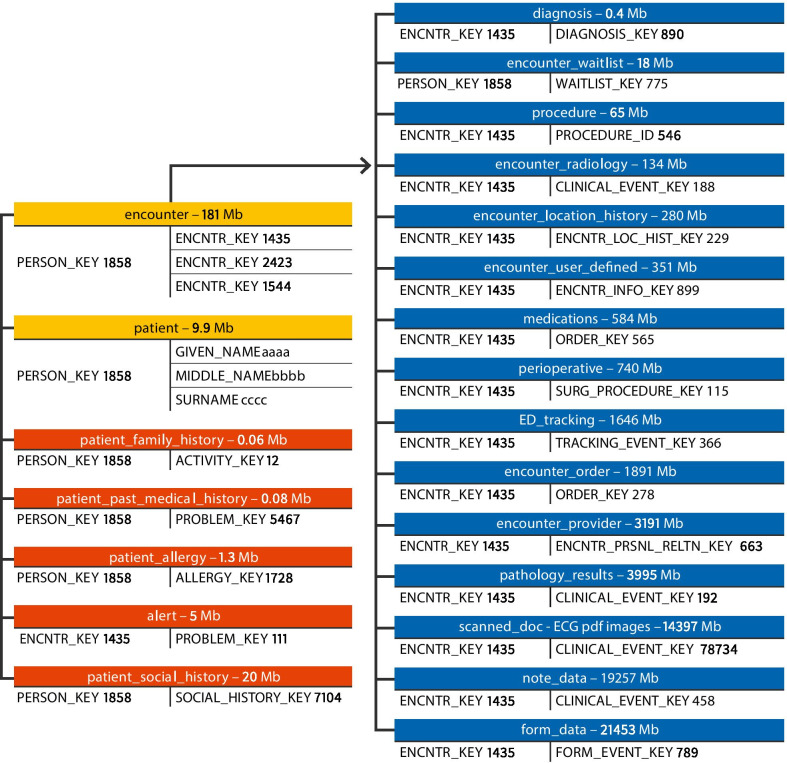
Visual representation of data tables extracted from Cerner Millenium EMR systems. Cerner EMR systems were extracted as data tables which are linked by encounter key. Data tables were linked by encounter key and include information about encounters, diagnoses (ICD-10 and SNOMED CT), pathology, forms (e.g. ED triage assessments, medication forms, discharge letters), notes (e.g. progress notes) and many more. Encounter level information were further linked to person level information by person key and included patient medical history and social history. The size of the raw file associated with each table is shown next to the table name. The data tables are listed in order of file size with the biggest files related to free-text information and scanned ECG images. The figure represents data extracted for a single 3 month time period (1/4/17–30/6/17)

Data extraction was performed for a single continuous five-year time period between 1/1/13 and 31/12/17. Encounters were extracted through the execution of a bespoke CCL script which contained seven functions (representing the seven inclusion criteria) which were designed to identify *eligible* encounters. Data extraction was performed by an external party (MKM Health, Chatswood, NSW) and in line with the HREC approval, the study investigators had no direct access to the EMR information systems or contained within the EMR. The external party wrote the CCL script which was then run on the production EMR system. Then, flat data tables were created, de-identified and extracted from the production EMR for the research team for analyses (Fig. 2). Approaches for ensuring data quality and the operational framework of the study (data management, security and governance) are described in Additional file 1: Information, Part 2.

### Outcome measures

EMR data extracts were processed and analysed using R (Version 4.0.0). Outcome measures included 1) examination of the relative contribution of individual inclusion criteria to the identification of eligible encounters, 2) associations between the use of diagnostic codes alone (ICD-10/SNOMED Clinical Terms (CT)) *and* other inclusion criteria and 3) examination of consistency across LHDs and time. First, we developed a computational method to check the composition of inclusion criteria met by each encounter. Each encounter received a score between 0 and 7 indicating how many of the inclusion criteria were met. Any encounter with a score ^3^1 was deemed eligible and included in the *index* cohort. Encounters that did not meet any of the inclusion criteria were assigned a value of ‘0’ and referred to as “*reference*” encounters (i.e. they are not eligible encounters). Next, we examined the proportions of encounters identified using diagnostic codes alone *vs*. other inclusion criteria, and vice-versa and a correlation matrix was used to calculate the associations between each of the inclusion criteria. Finally, we examined consistency in the composition of inclusion criteria across time and local health districts.

## Results

### Examination of the relative contribution of individual inclusion criteria to eligible encounters

The 5-year extract (1/1/13–31/12/17) consisted of 802,742 eligible and 5,418,466 reference encounters. Out of the 802,742 eligible encounters, scanned ECG images (54.8% of encounters) and symptoms and possible ACS-related keywords in clinical documentation (41.4–64.0%) were used most often to identify presentations of possible ACS. Orders and investigations (27.3%) and procedures (1.4%), were less often present for identified presentations. Relevant ICD-10/SNOMED CT codes were present for 3.7% of identified encounters.

**Fig. 3:**
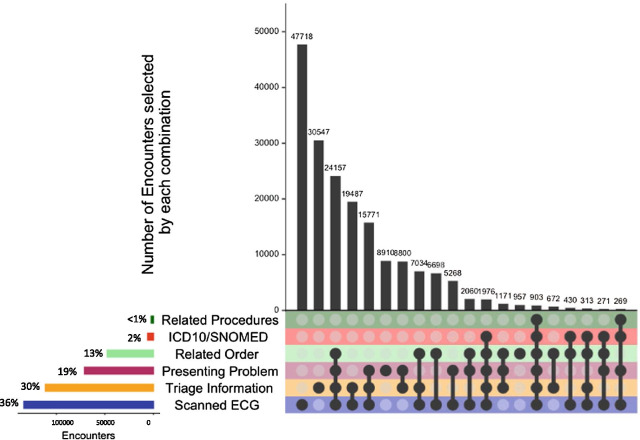
UpSet plot showing the number of encounters meeting individual (bottom left hand side) and multiple inclusion criteria (right-hand side). This UpSet plot represents 317,719 eligible encounters from Cerner information systems in two local health districts that met at least one of the study inclusion criteria in 2017. Inclusion criteria were described in Table 1. The histogram on the bottom left-hand side represents the total number and percentage of encounters that met each inclusion criterion. The plot on the top right-hand side represents the number and percentage of encounters that met each unique combination of inclusion criteria, depicted by the black circle(s) and lines. For example, the most frequent combinations were encounters that only had the presence of a scanned ECG image, followed by encounters that only had a keyword match in triage information

To further examine the composition of inclusion criteria in eligible encounters, UpSet plots [27] were used to represent the frequency of each inclusion criterion and the numbers of encounters that met each combination of inclusion criteria in 2017, the most recent data in our 5-year data extract. Figure 3 shows 185,414 eligible encounters in 2017 with similar findings as the total 5 year dataset. Individually, the presence of a scanned ECG image (72.0%), the presence of keywords captured in the presenting information in the ED triage form (60.0%) and the presence of keywords in the “Reason for visit” for the presentation (38.2%) identified the majority of eligible encounters. Orders and investigations (25.6%) and procedures (1.2%), were less often present for eligible encounters. Only 3.1% of encounters had the presence of a relevant diagnostic code (ICD-10 or SNOMED CT) for ACS. The presence of a cardiac monitoring form was not an informative criterion for identifying eligible encounters.

We found 59 unique combinations of inclusion criteria met by encounters (Fig. 3). The most frequent combinations were encounters that only had the presence of a scanned ECG image (26%; 47,514 of 185,414), encounters that only had a keyword match in triage information (18%; 33,865 of 185,414) and encounters that had the presence of an ECG, keyword match in triage information and a relevant order (12%; 22,160 of 185,414). Similar trends were seen in UpSet plots performed for the other years (2013–6; Additional file 2: Figure S1).

### Comparisons between diagnostic codes *and* other inclusion criteria

**Fig. 4:**
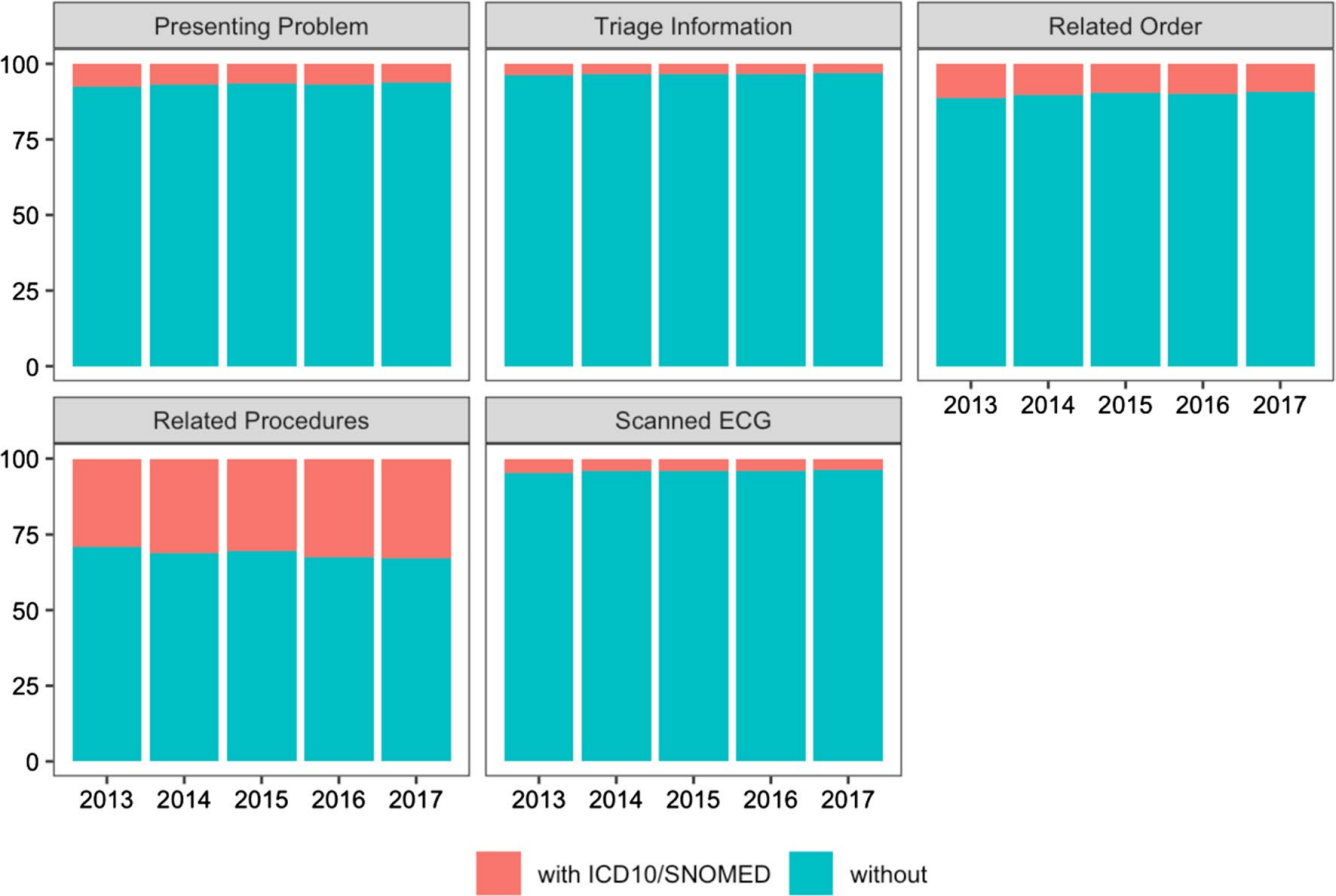
Percentage of eligible encounters with diagnostic codes (ICD-10/SNOMED CT) for each inclusion criteria, by year

In the 5-year extract, we examined the proportion of encounters that contained a relevant diagnostic code within each cohort of encounters met by each inclusion criterion. Given the broad inclusion criteria developed for this study, diagnostic codes comprised a minor component of each individual inclusion criteria cohort (Fig. 4). Note that 0.1% (858/802.742) of eligible encounters were identified using diagnostic codes alone. A correlation matrix examining associations between each of the inclusion criteria found a strong correlation between diagnostic code and relevant procedures (Pearson’s correlation coefficient = 0.9), with small to moderate associations with the other inclusion criteria (Additional file 3: Figure S2).

### Consistency across local health districts and time

**Fig. 5:**
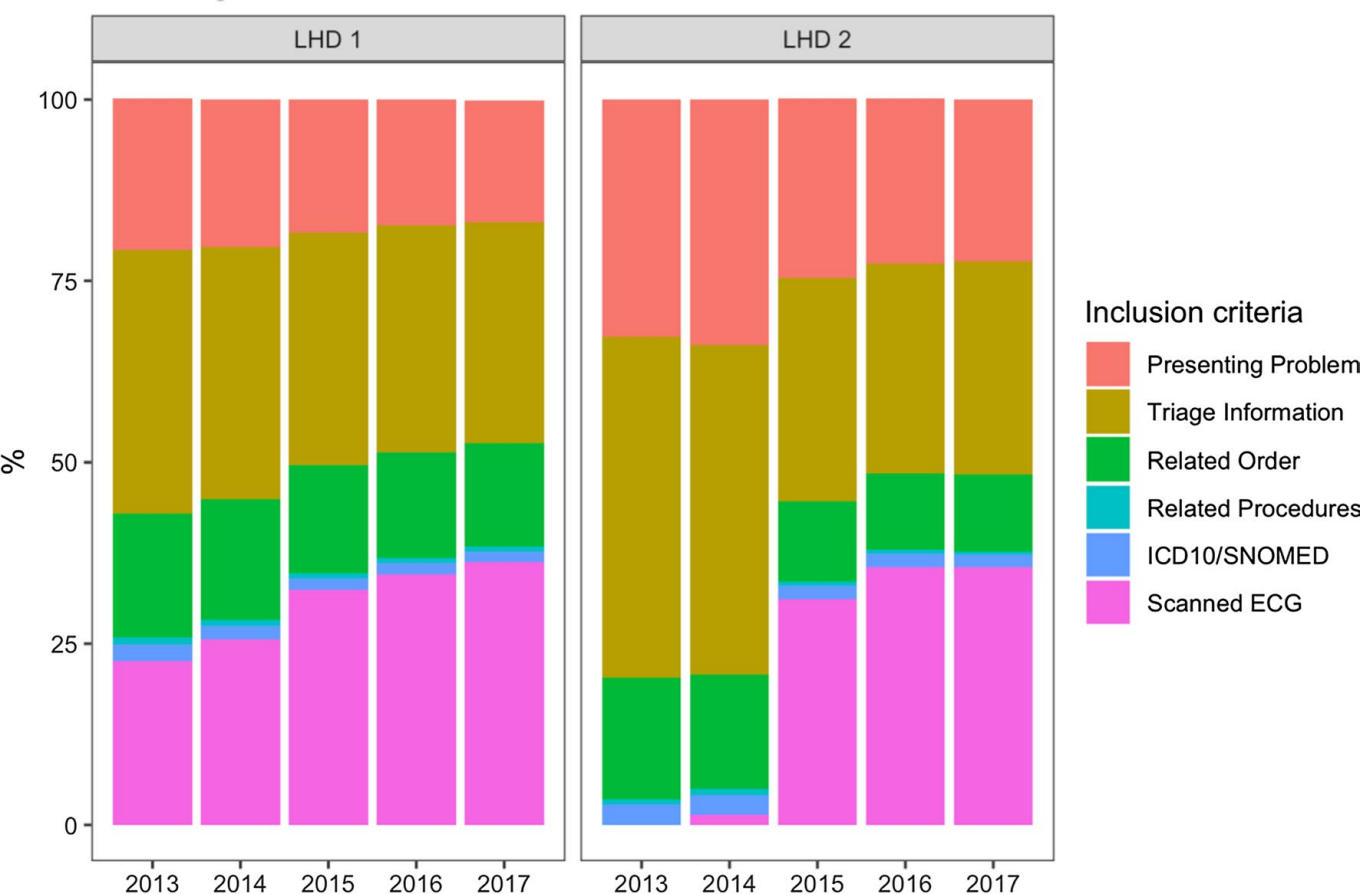
Percentage of total encounters in each local health district that met each inclusion criteria in a 5 year dataset. LHD 1 refers to Northern Sydney Local Health District and LHD 2 refers to Central Coast Local Health District.

To examine consistency of the methodology, we examined the frequency of each inclusion criterion for each year in our 5 year extract (Table 2). Individually, the presence of a scanned ECG image (15–36%), the presence of keywords captured in the presenting information in the ED triage form (19–25%) and the presence of keywords in the “Reason for visit” for the presentation (30–40%) identified the majority of eligible encounters. Orders and investigations (13–17%) and procedures (< 1%), were less often present for eligible encounters. 2–3% had the presence of a relevant diagnostic code (ICD-10 or SNOMED CT) for ACS. Similar trends were observed when the two LHDs were examined separately, with a diagnostic code being present in 2–3% of eligible encounters (Fig. 5).

**Table 2:** Percentage of encounters that met each inclusion criteria in the 5 year cohort

Year	Presenting problem, n (%)	Triage information, n (%)	Related order, n (%)	Related procedures, n (%)	ICD-10/SNOMED CT, n (%)	Scanned ECG image, n (%)
2013 (n = 135,511)	61,529 (45.4)	98,045 (72.3)	41,629 (30.7)	2296 (1.7)	6240 (4.6)	36,902 (27.2)
2014 (n = 143,108)	65,668 (45.8)	99,661 (69.6)	42,839 (29.9)	2259 (1.6)	5778 (4.0)	46,437 (32.5)
2015 (n = 163,008)	66,428 (40.8)	100,048 (61.4)	42,734 (26.2)	2099 (1.3)	5613 (3.4)	101,363 (62.2)
2016 (n = 175,701)	68,286 (38.9)	105,428 (60.0)	45,180 (25.7)	2353 (1.3)	5987 (3.4)	121,575 (69.2)
2017 (n = 185,414)	70,878 (38.2)	111,275 (60.0)	47,566 (25.7)	2243 (1.2)	5768 (3.1)	133,429 (72.0)

## Discussion

Our study demonstrates that informatics approaches combining structured EMR data, such as orders for pathology testing, investigations and diagnostic codes, with symptom and keyword text mining in narrative free-text during the data extraction process in production EMR systems, can create high fidelity clinical-defined patient cohorts. This inclusive approach to EMR data extraction enables subsequent datasets for EMR-derived cohorts to be readily created and updated as new conditions/diseases emerge and clinical definitions are updated [28], as well as the extraction of clinically-relevant information enabling future validation studies of diagnostic and procedure codes, which are essential for real-time clinical decision support and secondary use of EMR data [11, 29, 30]. This is particularly significant for diagnoses which have a diverse range of presenting problems (e.g. ACS, mental illness, sepsis) [31]. The use of diagnostic codes alone during this process would likely have led to relevant patients not being captured [32] as evidenced by the finding that < 1% of eligible encounters contained an ACS-related diagnostic code [29].

Our design approach for data extraction from the production EMR system balanced pragmatic and technological constraints against the broader study goal of creating a comprehensive EMR data platform that could be interrogated for a range of cardiovascular-related questions and other use cases in the future. For example, despite widespread use of regular expression and advances in natural language processing which would enable more thorough identification of presenting symptoms in free-text [33, 34], this was not available in the Cerner EMR implementation at the LHDs in this study. As such, these techniques were not able to be used production EMR systems, although there is progress to integrate them in the future [35]. Machine learning methods require access to specialist libraries and often high performance computing and tend to only able to be implemented *after* data has been extracted from production EMR systems. This study also served as a proof-of-concept for demonstrating to the organisation that data housed within EMR systems could be readily extracted for clinical utility.

Our work builds on earlier studies combining structured and unstructured free-text EMR data for identifying cohorts *after* data has been extracted from production EMR systems, demonstrating that diagnostic codes alone are insufficient for disease case detection. Penz et al. found that ICD-9 and current procedure terminology (CPT) codes identified less than 11% of the cases in a study of detecting adverse events related to central venous catheters, while natural language processing methods achieved a sensitivity of 0.72 and specificity of 0.80 [36]. Similar findings have been observed for detecting colorectal cancer cases [12]. Our study findings demonstrate that similar approaches for identifying cohorts are required within production EMR systems themselves, which have also been demonstrated by large collaborative research networks (e.g. PCORnet, eMERGE, ODHSI) [37].

The generalisable methodology described in this study is essential for curation of high-fidelity clinical data from EMR systems enabling continuous, routine monitoring and reporting on the quality of care and outcomes for an unselected cohort of patients across large health care treatment and referral networks or populations, as well as providing clinical decision-making guidance. Monitoring and reporting could be performed against agreed care standards and benchmarked outcomes for specific conditions, which would supplant the need for developing and maintaining labour intensive, condition-specific population-based observational cohort studies and clinical registries. A critical step of the methodology not fully described in this current paper is the requirement for validation studies comparing the clinically-defined EMR cohort definition against a gold standard to estimate sensitivity and specificity, prior to implementation into the production EMR. The gold standard depends on the context of how the EMR data will be used and could be clinician diagnosis if used for clinical decision support, or clinical registries or validation against ICD10 codes as previously performed in other conditions such as heart failure, post-traumatic stress disorder, Charleston Co-morbidity index, etc. [30, 38, 39]. A validation study estimating sensitivity and specificity for acute coronary syndrome comparing clinician diagnosis against ICD10 codes was performed in a 3 month EMR dataset from our current study; those results are currently under review [40].

The overarching strength of the research is the liberal nature of the data extraction process enabling future validation work critical for secondary use of EMR data, flexibility for creating new EMR-derived cohorts as clinical definitions and guidelines get updated as well as being able to identify and extract information on patients based on presenting symptoms and investigations, rather than diagnostic code. For example, using this generalisable methodology (Fig. 1) we can create EMR-derived cohorts for stroke, heart failure patients, etc. To our knowledge, this is also the first time that clinically-relevant information for diagnosing ACS has been collated from EMR data extracts (Additional file 1: Table S1). Limitations of the research include that the study period was restricted to extracted data from one information system (Cerner Millenium), chosen as it was the main EMR system, from only two of the fifteen local health districts in New South Wales and due to restricted access to the production EMR system, we were unable to iterate on the cohort identification process; this is possible when data extraction can occur on non-production EMR environments (i.e. clinical data warehouses) which are available at many sites in the UK and USA. Nevertheless the principles of this robust methodology can be applied to any EMR data extraction process and generalised to other diseases/conditions. Extending data extraction processes across health jurisdictions and for other conditions will enable further validation of the methodology.

### Conclusion

This paper demonstrates that clinically-defined EMR cohorts created using a broad strategy utilising structured and unstructured free-text in production EMR systems, are likely to identify relevant cohorts of patients and enable critical validation work required for real-time clinical decision support and secondary use of EMR data.

## Supplementary Information

**Additional file 1**. List of search terms for Inclusion Criteria 1 & 2, Approaches for ensuring data quality and study operational framework.

**Additional file 2: Fig. S1**. UpSet plot showing the number of eligible encounters meeting individual (bottom left hand side) and multiple inclusion criteria (right-hand side) in 2013–6. This UpSet plot represents eligible encounters from Cerner information systems in two local health districts that met at least one of the study inclusion criteria in 2013 (n = 135,511), 2014 (n = 143,108), 2015 (n = 163,008) and 2016 (n = 185,414). The bottom left-hand side represents the total number and percentage of encounters that met each inclusion criterion. Each inclusion criterion is represented as an independent group. The top right-hand side represents the number and percentage of encounters that met each combination of the inclusion criteria. Inclusion Criteria referred to: (1) *Presenting problem key match*: Keyword match in a free-text field for presenting information, (2) *Triage information key match*: The patient was assigned to a cardiac pathway mode of care or keyword match in the ED Triage descriptions, (3) *Related Order*: The existence of a cardiology-related order; (5) *Related Procedure*: the existence of cardiology-related procedure, (6) *ICD-10/SNOMED CT*: The encounter had an SNOMED CT or ICD-10 code for Acute Myocardial Infarction (AMI) and (7) *Scanned ECG image*: The encounter had a scanned ECG report available. Inclusion Criterion (4), representing patients that had a cardiac monitoring form, was excluded as no encounters met this inclusion criterion.

**Additional file 3: Fig. S2**. Correlation matrix examining associations between inclusion criteria.

## Data Availability

The datasets generated and/or analysed during the current study are not publicly available as they are owned by the Chief Executives of the Local Health Districts and not by the researchers who performed this study.

## Abbreviations

ACS: Acute coronary syndrome
CCL: Cerner Command Language
ECG: Elecrocardiogram
EMR: Electronic medical record
ICD-10: International Classification of Diseases and Related Health Problems (10th edition)
LHD: Local Health District
NSW: New South Wales
SNOMED CT: SNOMED clinical terms
STEMI: ST-elevation myocardial infarction
