# Supplementary material for: Combining structured and unstructured data in EMRs to create clinically-defined EMR-derived cohorts
Source: BMC Med Inform Decis Mak. 2021 Mar 8;21:91. doi: 10.1186/s12911-021-01441-w (PMC7938556; doi:10.1186/s12911-021-01441-w)
Supplement: Supplementary file 1 — Additional file 1. List of search terms for Inclusion Criteria 1 & 2, Approaches for ensuring data quality and study operational framework. [file 12911_2021_1441_MOESM1_ESM.docx]

**Supplementary Information**

## Supplementary Information Part 1:

The following provides a list of search terms which forms part of inclusion criteria (1):

”pain chest”|”pain,chest”|”pain-chest”|”chest pain”| “shortness of breath”| “sob”|”dizziness”|”vomiting”|”syncope”|”syncopal”|”presyncope”|”weakness”|”nausea”|”unwell”|”loc”|”cardiac arrest”| “nstemi”|”stemi”|”angio”|”angiogram”|”cor/angio”|”palpitation”|”salami”| “cabg”|”ami”|”coronary artery bypass graft”|”etami”|”heart attack”|”cath lab”| “cath”|”ohca”|”out of hospital arrest”| “out of hospital cardiac arrest”| “stent”| “fatigue”| “weakness”|”ventricular tachycardia”| “ventricular fibrillation”|”vt”|”vf”|”dyspnoea”|”chest tightness”

The following provides a list of search terms for inclusion criteria (2):

“ashen” |“chest pain”| “chest pains” | “chestpain”|“cp”|”chest tightness”| “clamminess”| “diaphoresis” | “diaphoretic”|”dizziness”|”dizzines” |”dizzy”|”dyspnoea”|”etami”|”fatigue”| “ingestion”|”indigestion”|”lightheadness”| “light headedness” |”lightheaded”| “light headed”| ”light-headed”|”loc”|”loss of consciousness”| “nausea”|”pale”| “palpitations”| ”palpatations”| ”palpations”|”palpitation”|”palpitaion”|”palpatation”|”shortness of breath”| “short of breath”| ”sob”| ”stemi”| ”nstemi”| ”sweaty”| ”sweats”| ”syncopal”| ”syncope”| ”syncopy”| ”syncople”| ”vomiting”|”vomiting”|”vomitting”|”vomting”|”weakness”|”ohca”|”vt”|”ventricular tachycardia”|”vf”|”ventricular fibrillation”|”clammy”|”presyncope”|”presyncopal”|”chest heaviness”|”epigastric pain”|”arm heaviness”|”failed thrombolysis”| “thrombolysis”

## Supplementary Information Part 2:

**Approaches to assessing data quality**

Data quality was assessed for completeness, accuracy and consistency [1].

*Completeness*

Completeness was assessed in several ways. Firstly, the percentage of missing values was ascertained for each data element. If data elements could be logically aggregated, then we examined the percentage of records with sufficient data to calculate an indicator or characteristic of interest (Eg. number of patients that had received a percutaneous coronary intervention). Ascertainment completeness, defined as the percentage of eligible cases present, was first compared by checking the face value of relevant characteristics with local clinicians. This was vital to develop confidence that all data elements for a required indicator had been identified and correctly extracted from the eMR. We also assessed the presence of duplicate records (Eg. diagnoses which were recorded twice), duplicate events (eg. a single procedure documented twice) and the absence of records (eg. All analytes which were measured in a single assay should have results recorded).

*Accuracy*

In the absence of a gold standard for assessing accuracy, or determining the true value of each record, we assessed validity, reliability, and as a theoretical step, the internal consistency of the encounters.

*Validity (internal and external)*

Internal validity was defined as the closeness of agreement between a data value and a plausible value. For pathology results, results were assessed against reference ranges. To verify time stamps, we checked that all time stamps fell within a patient’s admission and discharge time.

External validity was determined by checking distributions of variables and outliers, and comparing differences between our study, SPEED-EXTRACT and independent data sources published in similar clinical populations such as CONCORDANCE, a contemporary Australian observational registry that describes the management and outcomes in patients with ACS [2, 3] and another observational study of consecutive patients presenting with a ST-elevation myocardial infarction field triaged directly to the cardiac catheterisation laboratory within NSLHD [4]. Differences in results between SPEED-EXTRACT and published studies [2-4] were used to guide investigations as to whether data elements were missed during the data extraction process or characteristics were insufficiently captured. For example, smoking status can be collected as a structured field (eg. ex-, current- or non-smoker), however this was only filled in <1% of presentations, and tended to be collected in free-text clinical documentation.

*Reliability*

We examined relative uniformity in distributions of data elements across local health districts included in SPEED-EXTRACT (i.e., reproducibility) and temporal stability in the data extract across years at the same sites.

## Operational framework

*Data management and security*

The SPEED-EXTRACT project brought together expertise across local health districts, university and government agencies (ie. NSW Ministry of Health, eHealth NSW). In doing so, it navigated existing governance processes and created new processes to enable this work to occur across traditional organisational and professional silos. Information from the eMR was extracted and de-identified on secure servers within the local health district. The de-identified data was then securely transferred to the University Research Data Store server for storage and analyses. The University Research Data Store and the High Performance Computing cluster are University facilities located in Tier 3 data centres in Sydney with an Information Security Management system that conforms to the requirements of ISO27001:2013 international standard [5] and all relevant NSW laws, regulations and statutory requirements. Discrepancies between identifiable eMR data extracted within the local health district and de-identified eMR data received by the University were checked by designated employee(s) of the local health district as an additional data quality measure to those described above. All code developed on the de-identified data for the project was stored on a version control system.

*Project governance*

The project was directed by an Executive Committee comprising leaders in cardiology and digital health, population health and data science experts and representatives from government agencies. The Committee provided advice on content and scientific issues such as development of data definitions for computable phenotypes, understanding of local clinical workflow practices and interpretation of ACS quality and safety indicators. A number of subgroups devolved from the Executive Committee including a Data and Analytics Team and a Publication Committee. The Data and Analytics Team comprised software engineers, data analysts, data scientists and analytics translators and used agile project methodology to address the clinical questions set by the Executive Committee.

## Supplementary Table 1

## Data availability in SPEED-EXTRACT compared to existing cardiovascular eMR studies

| Data availability | SPEED-EXTRACT | CALIBER [6] | CANHEART [7] | CHERRY [8] |
| --- | --- | --- | --- | --- |
| Presenting symptoms | ✓ | X | X | X |
| ECG | ✓ | X | X | X |
| Troponin | ✓ | X | X | X |
| Clinical documentation | ✓ | X | X | X |
| Current and new medications | ✓ | X | X | X |
| Procedures | Codes and details | Codes only | Codes only | Codes only |
| Diagnoses | Codes and clinician diagnoses | Codes only | Codes only | Codes only |
| Clinical outcomes (In-hospital mortality, intensive care admission, length of stay, readmission rates) | ✓ | ✓ | ✓ | ✓ |
| Linkage to existing administrative datasets | Future work, readily able to be performed | ✓ | ✓ | ✓ |

**References**

1. [*https://rethinkingclinicaltrials.org/*](https://rethinkingclinicaltrials.org/). NIH Collaboratory Living Textbook of Pragmatic Living Trials 2020 1/2/20].

2. Aliprandi-Costa, B., et al., *The design and rationale of the Australian Cooperative National Registry of Acute Coronary care, Guideline Adherence and Clinical Events (CONCORDANCE).* Heart Lung Circ, 2013. **22**(7): p. 533-41.

3. Khan, E., et al., *Differences in management and outcomes for men and women with ST-elevation myocardial infarction.* Med J Aust, 2018. **209**(3): p. 118-123.

4. Vernon, S.T., et al., *Increasing proportion of ST elevation myocardial infarction patients with coronary atherosclerosis poorly explained by standard modifiable risk factors.* Eur J Prev Cardiol, 2017. **24**(17): p. 1824-1830.

5. <https://www.iso.org/standard/54534.html>. *ISO/IEC 27001:2013*. 2019.

6. Denaxas, S.C., et al., *Data resource profile: cardiovascular disease research using linked bespoke studies and electronic health records (CALIBER).* Int J Epidemiol, 2012. **41**(6): p. 1625-38.

7. Tu, J.V., et al., *The Cardiovascular Health in Ambulatory Care Research Team (CANHEART): using big data to measure and improve cardiovascular health and healthcare services.* Circ Cardiovasc Qual Outcomes, 2015. **8**(2): p. 204-12.

8. Lin, H., et al., *Using big data to improve cardiovascular care and outcomes in China: a protocol for the CHinese Electronic health Records Research in Yinzhou (CHERRY) Study.* BMJ Open, 2018. **8**(2): p. e019698.
