# Supplementary figures and images for: Combining structured and unstructured data in EMRs to create clinically-defined EMR-derived cohorts
Source: BMC Med Inform Decis Mak. 2021 Mar 8;21:91. doi: 10.1186/s12911-021-01441-w (PMC7938556; doi:10.1186/s12911-021-01441-w)

2013

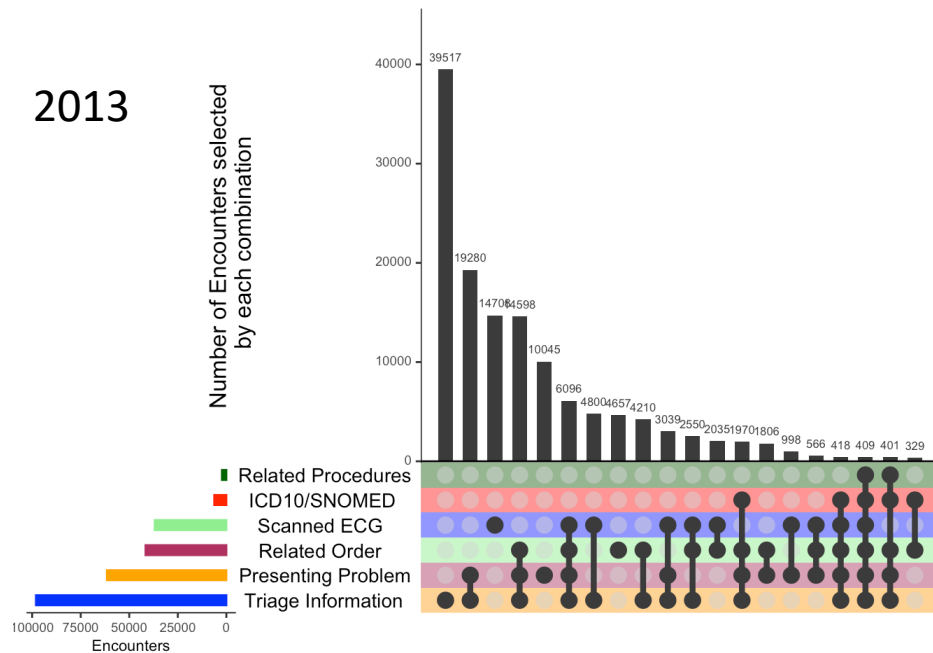

2014

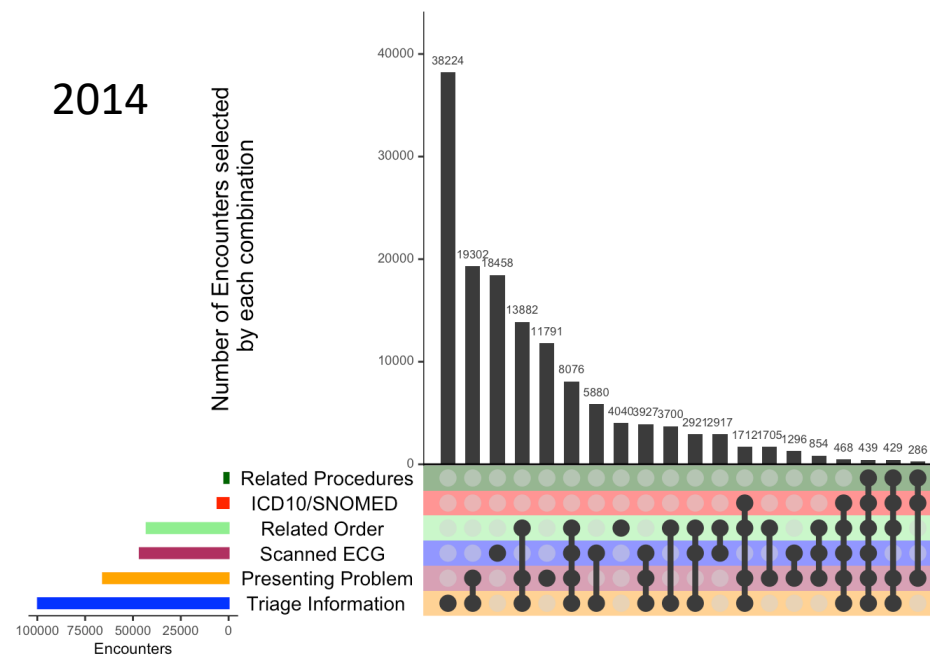

2015

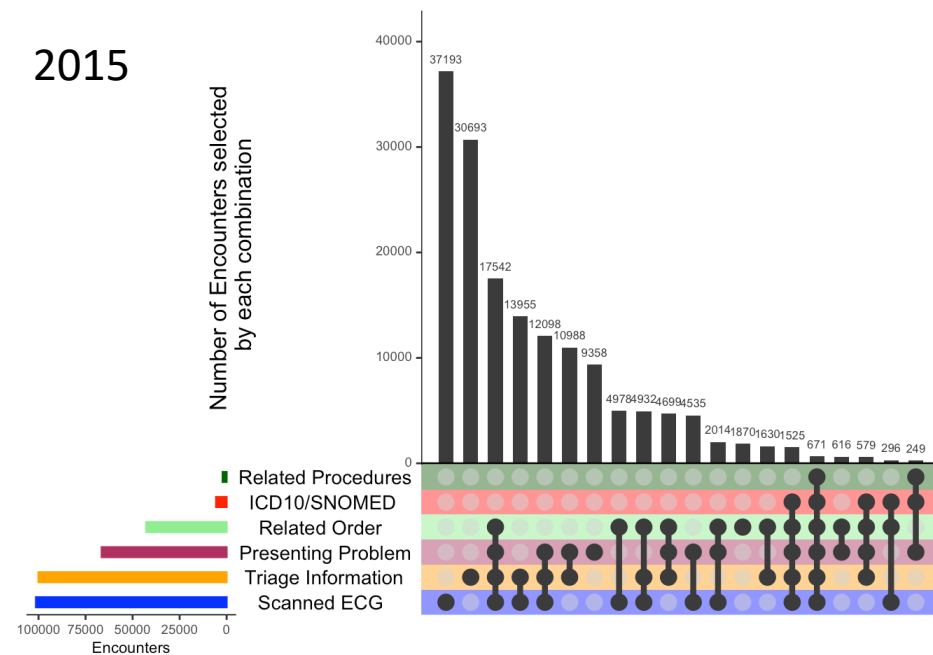

2016

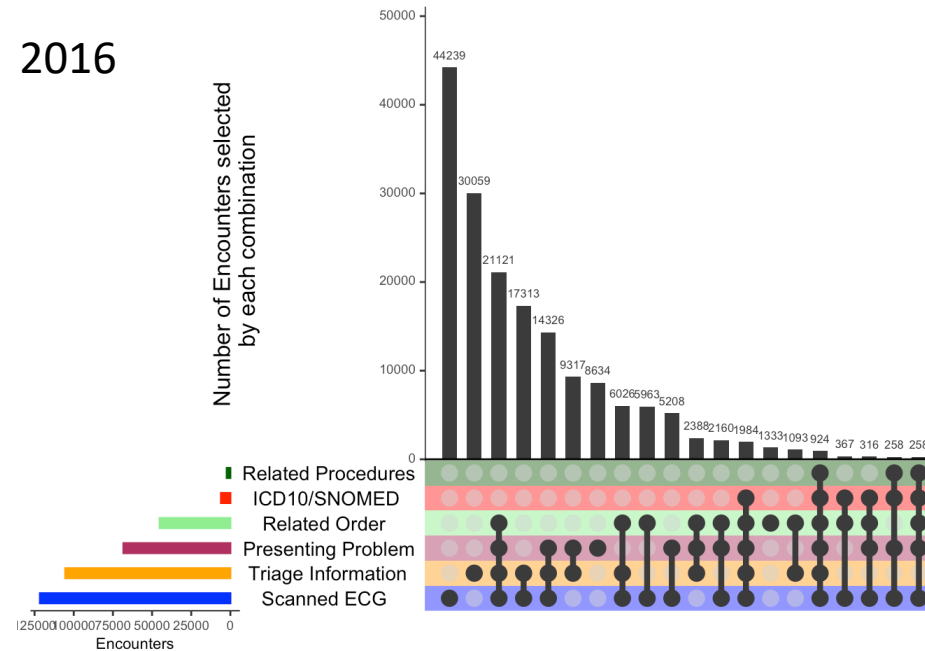

Supplement: Supplementary file 2 — Additional file 2: Fig. S1. UpSet plot showing the number of eligible encounters meeting individual (bottom left hand side) and multiple inclusion criteria (right-hand side) in 2013–6. This UpSet plot represents eligible encounters from Cerner information systems in two local health districts that met at least one of the study inclusion criteria in 2013 (n = 135,511), 2014 (n = 143,108), 2015 (n = 163,008) and 2016 (n = 185,414). The bottom left-hand side represents the total number and percentage of encounters that met each inclusion criterion. Each inclusion criterion is represented as an independent group. The top right-hand side represents the number and percentage of encounters that met each combination of the inclusion criteria. Inclusion Criteria referred to: (1) Presenting problem key match: Keyword match in a free-text field for presenting information, (2) Triage information key match: The patient was assigned to a cardiac pathway mode of care or keyword match in the ED Triage descriptions, (3) Related Order: The existence of a cardiology-related order; (5) Related Procedure: the existence of cardiology-related procedure, (6) ICD-10/SNOMED CT: The encounter had an SNOMED CT or ICD-10 code for Acute Myocardial Infarction (AMI) and (7) Scanned ECG image: The encounter had a scanned ECG report available. Inclusion Criterion (4), representing patients that had a cardiac monitoring form, was excluded as no encounters met this inclusion criterion. [file 12911_2021_1441_MOESM2_ESM.pdf]

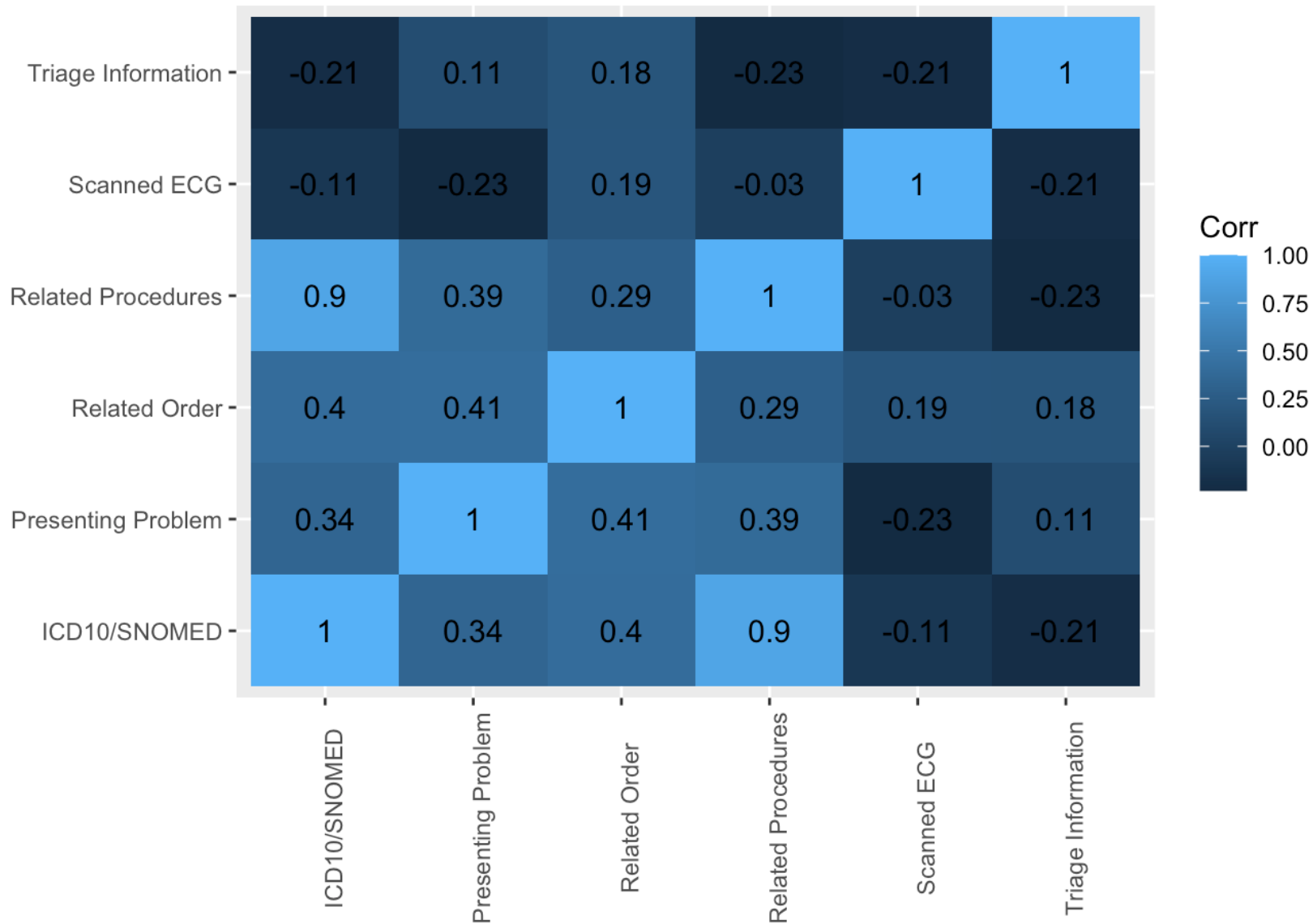

Supplement: Supplementary file 3 — Additional file 3: Fig. S2. Correlation matrix examining associations between inclusion criteria. [file 12911_2021_1441_MOESM3_ESM.pdf]
